# A Fiber-Optic Surface Plasmon Resonance Sensor for Bio-Detection in Visible to Near-Infrared Images

**DOI:** 10.3390/bios12010009

**Published:** 2021-12-23

**Authors:** Shimeng Chen, Haojun Wu, Yongxin Song, Wei Peng, Yun Liu

**Affiliations:** 1 Department of Marine Engineering, Dalian Maritime University, Dalian 116026, China; dlhsdxwhj@dlmu.edu.cn (H.W.); yongxin@dlmu.edu.cn (Y.S.); 2 School of Physics, Dalian University of Technology, Dalian 116024, China; wpeng@dlut.edu.cn

**Keywords:** fiber optic, surface plasmon resonance, biosensor, image detection

## Abstract

In this paper, we demonstrate a fiber-optic surface plasmon resonance (FO-SPR) biosensor based on image processing and back propagation (BP) neural network. The transmitted light of the FO-SPR sensor was captured by using visible (VIS) and near-infrared (NIR) CMOS sensors. The optical information related to the SPR effect was extracted from images based on grayscale conversion and an edge detection algorithm. To achieve accurate monitoring of refractive index (RI) changes, the grayscale means of the VIS and NIR images and the RGB summation of the edge-detected images were used as training and test inputs for the BP neural network. We verified the effectiveness and superiority of this sensing system by experiments on sodium chloride solution identification and protein binding detection. This work is promising for practical applications in standardized biochemical sensing.

## 1. Introduction

Surface plasmon resonance effect (SPR) is a free-electron resonance oscillation on the metal surface, which is excited by an incident light at the interface of metal and dielectric [1,2,3]. Since Nylander and Liedberg applied the SPR sensing technology to gas detection and biosensing for the first time in 1983 [4], SPR technology has been widely used in medical diagnosis [5], biological detection [6], environmental pollution detection [7], food safety testing [8], and other fields [9]. Because refractive index (RI) is the inherent characteristics of most biomaterials and SPR effect is very sensitive to RI change of a medium attached to metal film [10], it makes the SPR-based biochemical analysis technique possible to detect biofluid by perceiving a small RI variation. Due to the kinds of advantages, such as label-free, high sensitivity, and rapid detection, companies have produced some commercial SPR instruments in recent years [11,12,13]. However, these SPR biological sensing systems were designed for the laboratory instruments that were generally bulky, expensive, and time consuming [14,15]. Generally, resonance angle, resonance wavelength, reflection intensity, and phase information can be used as the sensing signals of SPR instruments [16,17,18,19]. The demodulation methods based on resonance angle and wavelength are relatively reliable while the SPR instruments demodulated by these two methods are hard to be miniaturized and are high in cost. The phase information is highly susceptible to external interference, although the phase-based SPR instruments have a high sensitivity. The intensity detection method is not as accurate as the former, but it has advantages in low cost and easy miniaturization. Most of the SPR instruments built by the laboratory adopted an angular scanning method and wavelength modulation, but the spectrometer and rotary plates driven by the stepper motor led to rising cost and volume of the SPR instruments [20,21].

Therefore, the development of miniaturized and low-cost SPR sensing devices is of great significance to the applications of SPR sensing technology [22,23,24]. The main purpose of this work was to achieve cost-effective and compact SPR sensing. Our previous work proposed a biochemical sensing method by monitoring the light intensity in the fiber end face [25]. The self-compensated scheme of the sensing method used different optical fibers as measurement and reference channels. However, because these optical fibers were not identical, there are more or less differences in optical properties, geometric dimensions, and illuminations among them. Therefore, it is hard to completely eliminate the effects of power fluctuation of a light source by comparing the reference channel with the measurement channel. Our other research showed that different areas on the end face of the same capillary sensing element can be selected as measurement and reference channels, which can effectively avoid the error caused by the inconsistency of optical fibers [26]. However, the processing operation involves multiple chemical treatments in different areas on the surface of capillary, which is much more complicated than the optical fiber element.

This paper proposes a simple, self-compensated biochemical sensing method based on a single fiber-optic SPR (FO-SPR) sensor and back propagation (BP) neural network. We used visible (VIS) and near-infrared (NIR) CMOS image sensors to capture the image of transmitted light from the FO-SPR sensing element. We applied the grayscale conversion and edge detection algorithms to the original images. Then, the information of brightness and shape was employed as inputs of the BP neural network for training and testing to predict the specific RI value of the sample. The experimental results showed that we realized the accurate detection of RI and biochemical samples without the need for a compensation mechanism to prevent the fluctuations in light source intensity.

## 2. Materials and Methods

### 2.1. Materials and Reagents

Immunoglobulin G (IgG) from rabbit serum, anti-rabbit IgG antibody produced in goat and phosphate buffer solution (PBS, pH 7.4) were purchased from Sangon Biotech Co., Ltd. (Shanghai, China). The 1-(3-Dimethylamino-propyl)-3-ethylcarbodiimide hydrochloride (EDC) and N-Hydroxysuccinimide (NHS) were purchased from Saan Chemical Technology Co., Ltd. (Shanghai, China). Bovine serum albumin (BSA), 11-mercaptoundecanic acid (MUA), and other chemicals were purchased from Sigma-Aldrich Trading Co., Ltd. (Shanghai, China). All of the reagents were of analytical grade and could be used without further purification. All proteins were dissolved into PBS before use.

### 2.2. Fabrication of FO-SPR Sensing Element

We fabricated the FO-SPR sensing elements with the plastic-cladded silica optical fiber (HP 400/430-37/730E YOFC; core and cladding diameters of 400 and 430 µm, length of 3 cm). The plastic coating and cladding of the fiber were stripped to 1 cm in length in the center. After washing and drying, the fiber was coated with chromium/gold layers (Cr 5 nm, Au 50 nm) using a magnetron sputtering system (K575XD from E.M. Technologies Ltd. Ashford, Kent). All end faces of the fiber were polished with emery paper. The FO-SPR sensing element was then packaged in a flow cell.

### 2.3. Anti-Body Immobilization

The gold film surface of the FO-SPR sensor was rinsed with ethanol and water and dried with nitrogen. Then, the sensor was soaked in a 1 mM 11-mercaptoundecanic acid (MUA) in ethanol for 12 h and rinsed with ethanol. Afterwards, a mixed NHS (0.5 M) and EDC (0.5 M) aqueous solution was introduced for 15 min at 4 °C to form a reactive ester molecular layer for activation. Then, we introduced the PBS buffer containing anti-rabbit IgG (0.2 mg/mL) into the flow-cell and let it stand for 30 min. The protein molecule could be immobilized on the activated surface of gold film. Then, the FO-SPR sensing probe was rinsed with PBS buffer and 1 mg/mL BSA solution was injected into the flow-cell to block the unbound site.

### 2.4. Sensor Interrogation

**Figure 1:**
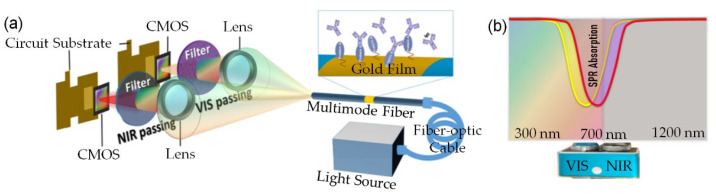
(**a**) Schematic diagram of a dual-camera FO-SPR sensing device; (**b**) principle of detecting the change of SPR absorption by VIS and NIR cameras. (The yellow and the red lines, respectively, represent the spectrum of the RIs of the external environment from small to large.)

The sensing setup is shown in Figure 1. A dual-lens camera containing two CMOS image sensors for VIS and NIR waveband (640 × 480 resolution, 15 fps) was used as a detector. Broadband light from a halogen lamp (with adjustable brightness and a wavelength range of 360–2000 nm) was coupled into a FO-SPR component through an optical fiber cable. Transmission light was detected by VIS and NIR CMOS sensors at the same time. The CMOS sensors were equipped with the filters having spectral ranges of 300–700 nm and 700–1200 nm. The bit depths were 24 bits and 10 bits, respectively. 

### 2.5. Machine Learning Algorithm

The BP neural network is a multi-layer feedforward neural network, which consists of three layers, including an input layer, a hidden layer, and an output layer in our experiment [27]. In this work, the number of input neurons was set as 4, the number of neurons in the hidden layer was set as 10, and the number of neurons in the output layer was set as 1 to represent the corresponding refractive index values.

## 3. Results and Discussion

### 3.1. SPR Image Processing and Sensing 

**Figure 2:**
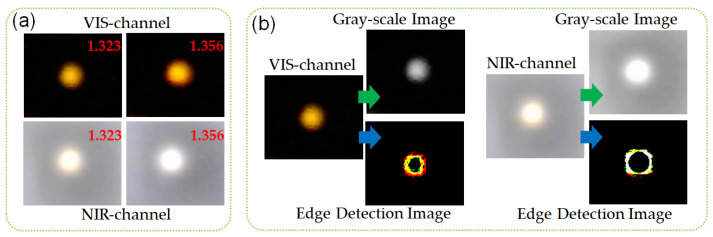
(**a**) Experimentally measured transmissions of optical fiber SPR sensors at RIs of 1.323 and 1.356; (**b**) VIS and NIR images of fiber end face converted into grayscale and edge detection images.

For the traditional SPR image sensor, VIS images are usually collected for data analysis. However, the FO-SPR sensing element can achieve an obvious energy absorption of SPR (corresponds to a transmittance of less than 100%) covering a wide wavelength range (spanning under the VIS and NIR waveband) when an external medium is an aqueous solution, as shown in Figure 1b. Therefore, the change of light intensity in the VIS band (the operating wavelength range of VIS CMOS is 300–700 nm) and NIR band (the operating wavelength range of NIR CMOS is 700–1200 nm) can reflect the energy absorption characteristics of SPR. Both VIS and NIR CMOS sensors can perceive the absorption area. The RI fluctuation of aqueous solution had a distinct influence on transmission of VIS and NIR light from FO-SPR sensor. Therefore, the images collected by NIR CMOS sensors were introduced for improving sensing performance. In contrast to our previous work on fiber-optic surface plasmon resonance (FO-SPR) image sensing technique [25,28,29], the relation between the grayscale mean value of our sensor’s light spot image and the RI was neither a linear nor monotone function. Therefore, we could not just determine RI according to the grayscale mean value alone. This was because the grayscale mean value depends on three variable factors at the same time: the RI variation, the stability of the broadband light source, and the absorption characteristics of SPR spectrum. For achieving RI measurement, the application of BP neural network in multivariate calibrations was proposed in our work to find the complex nonlinear relation between the RI and the light spot image information. For our FO-SPR sensing system in VIS-NIR images, we chose four characteristic values, including the grayscale mean and RGB summation of the edge detection images of the VIS and NIR light spot images, as shown in Figure 2. 

The grayscale mean value was chosen as the input layer because it had the light intensity information by VIS and NIR COMS sensors for the light spot images. It was closely related to the SPR absorption. RGB summation of the edge detection image was chosen as the input layer because the diameters of the light spot were different when the COMS sensors collected the light spot images with different light intensities. The profile of the light spot edge was closely related to the energy distribution of SPR mode in the fiber. The diameter of the light spot was simultaneously affected by the performance of the light source and COMS sensor, as well as the supersaturation condition. Thus, more accurate RI information can be determined by collecting grayscale mean value and RGB summation of the edge detection image simultaneously. In order to verify the feasibility of the above design scheme, we conducted preliminary testing on the proposed sensing device. We adopted two aqueous solutions with RI values of 1.323 and 1.356 as test samples. The corresponding light spot of the FO-SPR component recorded by VIS and NIR CMOS image sensors are shown in Figure 2a. When the RI value increased from1.323 to 1.356, we found that both the VIS and the NIR images had a slight change in the brightness and shape of the light spot. This result indicated that the information about RI fluctuation was included in the images of the transmission light from fiber sensor. As a result, we could determine the change in RI through multi-source information extraction of VIS and NIR light from FO-SPR sensor. This was helpful to obtain a robust result. The proposed method for RI monitoring contained three steps. First, VIS and NIR images of the end face of the fiber SPR sensor were recorded by the CMOS image sensors and processed by grayscale conversion and the edge detection algorithm, as shown in Figure 2b [30,31]. Gray scales were used to represent the proportion of “red, green, and blue” in the color image. To get a grayscale image, the color information from each channel was removed, leaving only the luminance values. In this work, we calculated the grayscale by using floating point arithmetic: Grayscale = R × 0.3 + G × 0.59 + B × 0.11. Then, the mean values of the grayscale images and the RGB summation values of the edge detection images were calculated to be used as conditional indicators. Finally, the mean values and RGB summation values were used as inputs of the BP neural network, while the output of the BP neural network represented a different RI response. In this work, the image processing and BP neural network implementation were based on Matlab software.

### 3.2. Response to RI Variation of Aqueous Solution

**Figure 3:**
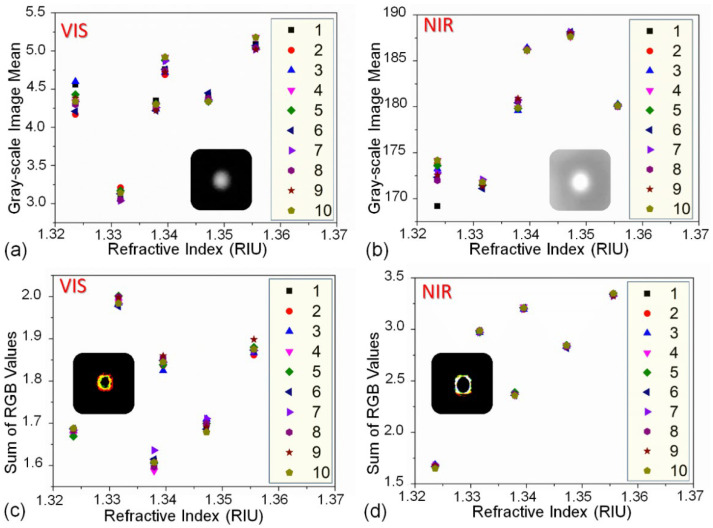
Grayscale mean of the VIS (**a**) and NIR (**b**) light spot images at different RIs. RGB summation of the edge detection images of VIS (**c**) and NIR (**d**) light spot at different RIs.

During the experiment, we recorded the images of the fiber end faces when the FO-SPR sensor was tested by sodium chloride solutions with RI values ranging from 1.323 (ultra-pure water) to 1.356 (12% concentration). Ten images were recorded with an interval of 5 min for each solution. The processed data from these images are shown in Figure 3. The data points in Figure 3a represent the grayscale mean values of VIS images at six different RIs. The power fluctuation of the light source caused some influence that made it impossible to infer the RIs effectively. For example, the distribution of different data points for the grayscale mean of the same RI did not completely coincide for VIS images. In addition, for different RIs (such as 1.323, 1.338, 1.347), the grayscale mean values were almost the same; therefore, we also could not distinguish their grayscale mean values of VIS images. Therefore, it was necessary to introduce other characteristic values. The data points in Figure 3b represent the grayscale mean of NIR images at six different RIs. The data points in Figure 3c,d represent the RGB summation of the edge detection images calculated using the Prewitt operator at six different RIs. (Prewitt operator is a discrete differentiation operator, which is based on convolving the image with a small, separable, and integer-valued filter in horizontal and vertical directions. It detects the edge by using the principle of the grayscale difference between the neighboring pixel points reaching the maximum value at the edge.) Comparing the RGB summation values of the edge detection images of the VIS and NIR images, it can be found that the data points of the NIR image had better coincidence (for the same RI). This reason may be the fact that the NIR light spot was much brighter than VIS light spot. The shape of the VIS light spot was greatly affected by the fluctuations in the light source power. Additionally, the boundary was relatively fuzzy and unstable.

**Figure 4:**
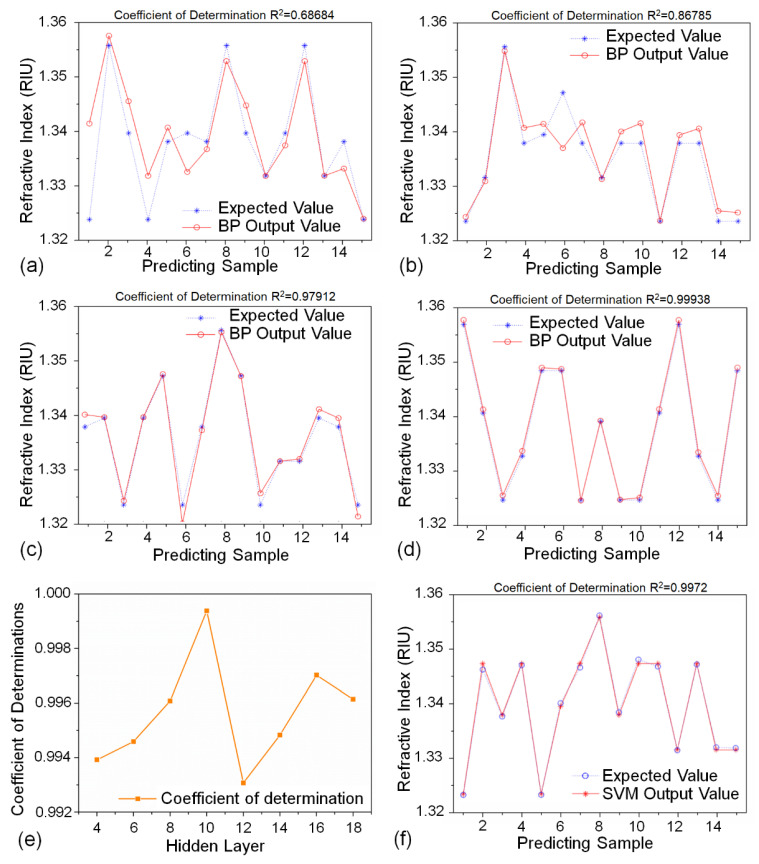
(**a**) BP neural network result by using the grayscale mean of VIS images as input; (**b**) BP neural network result after adding the grayscale mean of NIR images as input; (**c**) BP neural network result by using the grayscale means of VIS and NIR images and RGB summation of the VIS edge detection images as input; (**d**) BP neural network result after adding RGB summation of the NIR edge detection images as input. (**e**) Coefficient of determination when the number of neurons in hidden layers is 4, 8, 10, 12, and 14; (**f**) SVM result by using the grayscale means of VIS and NIR images and RGB summation of the VIS edge detection images as input.

In order to avoid the influences of different data sets when verifying the proposed method, we selected 45 data sets from all 60 data sets as training sets. The remaining 15 data sets were determined as test sets. The test sets were randomly characterized in this work. We randomly divided 45 training sets and 15 test sets into a total of 60 data sets each time. The expected values in Figure 4 are the RIs of the sodium chloride solution samples flowing into the flow cell during the experiments. The RIs of the samples were measured by an Abbe refractometer. Besides, to explore the effects of the four types of input (the grayscale means of VIS and NIR images and RGB summation of the edge detection images) on the BP neural network output, we used only the grayscale mean dataset of VIS images as the input to the BP neural network firstly. The BP neural network results are shown in Figure 4a–d where the abscissa represents the test data set and the ordinate represents the corresponding RI value. We calculated R^2^, the coefficient of determination, as an indicator of the difference between the test results and the expected results. As can be seen from Figure 4a, the coefficient of determination was 0.68684. This indicated that the recognition result was unsatisfactory and the results of the training and testing process were disordered. The reason is that the grayscale mean of the VIS images did not contains sufficient characteristic information on the corresponding RI. Therefore, we added the grayscale mean of the NIR channel image as the input to the BP neural network. The test results are shown in Figure 4b. We can see that the value of the decision coefficient increased to 0.86785. Compared with the results of Figure 4a, the accuracy of recognition was significantly improved. Thereafter, we further added the RGB summation values of the VIS and infrared edge detection images as input to the BP neural network. The test results are shown in Figure 4c. It can be found that the recognition accuracy of the BP neural network test set using three types of input (VIS and NIR channel image grayscale means, and VIS RGB summation of the channel edge detection image) was 0.97912, which is much higher than the previous results, shown in Figure 4a,b. In addition, as shown in Figure 4d, the BP neural network test set with all four types of input had a recognition accuracy of up to 0.99938, which can almost completely predict the RI value represented by the test data set. Thus, the above experimental results demonstrated the correctness and effectiveness of our method for monitoring the signal of FO-SPR sensor.

**Figure 5:**
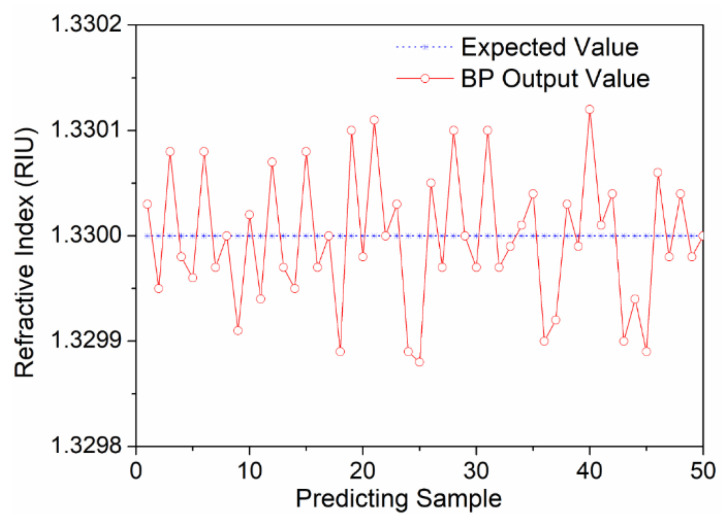
BP neural network result obtained by using the grayscale means of VIS and NIR images and RGB summation of the VIS and NIR edge detection images as input. The RI of the measured sample remained constant.

Besides, we also set the number of neurons in hidden layers to 4, 8, 12, and 14 to investigate the effect of this parameter on the performance of the neural network. The coefficient of determination for each number is shown in Figure 4e. We found there was a least deviation between the output value and the expected value when the number of neurons in hidden layers was 10. To compare the performance of the BP neural network with other machine learning algorithm, we also used the support vector machine (SVM) algorithm and optimized its parameters using the grid search method. Figure 4f shows the comparison of the output result with the expected result. The coefficient of determination was 0.9972, which was slightly lower than that of the BP neural network model. To sum up, the four characteristic values were set as the input layer and the hidden layer was set as 10 in the following experiments. The stability of our sensing system was tested to assess noise level, as shown in Figure 5. The resolution of our system was 6.3 × 10^−5^ RIU. The proposed sensing system can better solve the noise problem of the SPR image sensor. The BP neural network model can be used to effectively predict the nonlinear relationship between the RIs and the SPR signals affected by noise. Therefore, our sensing system had good effectiveness and robustness.

### 3.3. Response to Antigen–Antibody Binding

**Figure 6:**
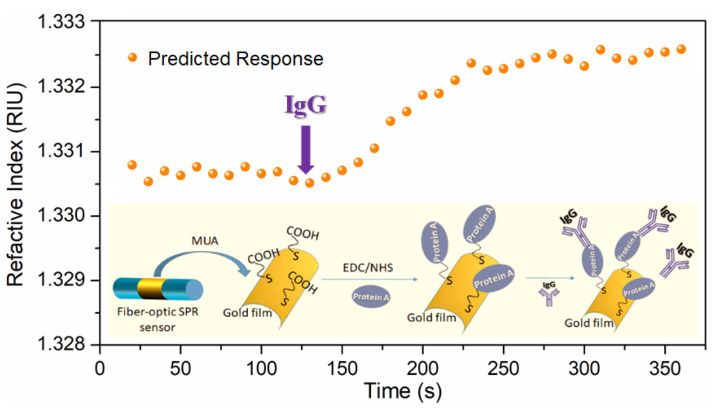
Predicted response of IgG samples flowing through the sensing surface by using BP neural network. Inset: Functionalization process on gold film of FO-SPR component.

In order to use this sensing device for biosensing, we functionalized the FO-SPR sensor. As shown in the inset of Figure 6, the probe biomolecules were immobilized on the surface of the gold film by self-assembly. When an aqueous sample was introduced and flowed over the gold film, the gold surface modified with an antibody molecular layer could specifically capture antigen molecules. This caused a local RI change near the gold surface and influenced the surface plasmon waves. We prepared an IgG antibody solution of 0.1 mg/mL. The PBS buffer and IgG solution were sequentially introduced into the flow cell. The flow cell was fabricated using centrifuge tubes of 0.2 mL via typical drilling and cutting processes. Sample solutions were allowed to flow through the flow cell at a flow rate of 0.2 mL/min via a peristaltic pump (BT200-2J). We recorded the images of the fiber end face every 10 s. Both of VIS and NIR channel images were processed by grayscale conversion and an edge detection algorithm. We extracted the grayscale mean and RGB summation of the processed images as before. Then, we used these data sets as test sets of the trained BP neural network and obtained the predicted RI response. The relation between the predicted RI values of the output and the time is shown in Figure 6. A significant signal response after IgG injection was found. This corresponded to the binding process between anti-IgG and IgG on the surface of the gold film. Such a result indicated that our method can monitor the binding of protein on the sensing surface in real time.

The limit of detection (LOD) of biomolecules is one of the important parameters; so, we calculated the LOD of our sensing system first. LOD was calculated using the method recommended by IUPAC in the Journal of Analytical Chemistry [32,33], which is defined as the concentration corresponding to triple standard deviation of sensing system background noise. Since the BP neural network model did not need to fit the response to obtain the sensitivity, we could not calculate the LOD through the sensitivity. According to the basic principle of SPR technique, the reason why we can detect the specific binding of proteins is that the biomolecule binding process can cause the RI variation on the SPR sensing surface. Based on our previous work [28], the RI variation caused by the binding process of IgG molecule on the gold film surface had an approximate linear relationship with the concentration of IgG (small concentration, far lower than the saturation concentration). According to our previous experimental result, The RI of the sensing surface can be changed by 10-5 RIU when the IgG concentration changes by 46 pM. Therefore, the LOD of our sensing system was calculated as 0.9 nM based on our stability test above.

### 3.4. Comparison

As shown in Table 1, we compared some miniaturized SPR devices in terms of resolution and LOD in terms of resolution and LOD. In addition, we used a miniaturized commercial SPR equipment to detect an IgG-specific binding process. In order to better compare LOD of IgG, we unified the concentration units in the table. According to the comparison results, our sensing system had good effectiveness and superiority.

## 4. Conclusions

This paper demonstrated a biosensing method based on image feature analysis and BP neural network. By using VIS and NIR CMOS sensors to capture the transmitted spot image of the fiber SPR sensor, the grayscale signal conversion and edge detection algorithms were used to extract the optical signals from images of a fiber end face, which contained the information of RI fluctuation near the sensing surface. We used the grayscale mean of the VIS and NIR images and the RGB summation of the edge detection images as training and test sets for the BP neural network to achieve an accurate and stable RI response monitoring. We verified the effectiveness and superiority of our method by experimental studies on different sodium chloride solution and protein binding detections. This method can be further investigated to monitor more FO-SPR sensors to achieve a multi-channel biochemical sensing. 

## Figures and Tables

**Table 1:** Comparison of the sensing performance for a small-sized SPR sensor.

Strategy	Resolution	LOD of IgG	Ref.
Chemically etched, single-mode fiber probe	1.6 × 10^−3^ RIU		[34]
Dual-Channel SPR System	4.3 × 10^−4^ RIU		[29]
Reflectance-based disposable fiber probe	9.3 × 10^−5^ RIU		[35]
Smart phone SPR biosensor	7.4 × 10^−5^ RIU	47.4 nM	[28]
MMF-NCF-MMF structure		11.7 nM	[36]
A polydopamine-modified FO-SPR biosensor		6.0 nM	[37]
Ag nanocubes/chitosan composite		4.0 nM	[38]
Multimode-coreless-multimode fiber probe		3.1 nM	[39]
Miniature commercial SPR instrument	2.7 × 10^−5^ RIU	15.7 nM	**Biosuplar6**
FO-SPR based on (BP) neural network	6.3 × 10^−5^ RIU	0.9 nM	**This work**

## Data Availability

Not applicable.
